# The Role of Curcumin in Cancer: A Focus on the PI3K/Akt Pathway

**DOI:** 10.3390/cancers16081554

**Published:** 2024-04-18

**Authors:** Vasiliki Zoi, Athanassios P. Kyritsis, Vasiliki Galani, Diamanto Lazari, Chrissa Sioka, Spyridon Voulgaris, Georgios A. Alexiou

**Affiliations:** 1 Neurosurgical Institute, University of Ioannina, 45500 Ioannina, Greece; 2 Department of Anatomy Histology-Embryology, School of Medicine, University of Ioannina, 45500 Ioannina, Greece; 3 Laboratory of Pharmacognosy, Faculty of Health Sciences, School of Pharmacy, Aristotle University of Thessaloniki, 54124 Thessaloniki, Greece; 4 Department of Neurosurgery, University of Ioannina, 45500 Ioannina, Greece

**Keywords:** curcumin, PI3K, glioblastoma, cancer

## Abstract

**Simple Summary:**

Cancer has a major impact on societies across the world. In an attempt to find new treatment options for cancer, attention has shifted to natural compounds. Curcumin is a polyphenol isolated from the roots of turmeric that possesses many biological properties. It acts on the regulation of different aspects of tumor development and interconnects with major signaling pathways that are dysregulated in cancer, such as the phosphatidylinositol-3-kinase/protein kinase B pathway. In this review, the diverse effects of curcumin on the regulation of this pathway in different malignancies will be discussed.

**Abstract:**

Cancer is a life-threatening disease and one of the leading causes of death worldwide. Despite significant advancements in therapeutic options, most available anti-cancer agents have limited efficacy. In this context, natural compounds with diverse chemical structures have been investigated for their multimodal anti-cancer properties. Curcumin is a polyphenol isolated from the rhizomes of *Curcuma longa* and has been widely studied for its anti-inflammatory, anti-oxidant, and anti-cancer effects. Curcumin acts on the regulation of different aspects of cancer development, including initiation, metastasis, angiogenesis, and progression. The phosphatidylinositol-3-kinase (PI3K)/protein kinase B (AKT) pathway is a key target in cancer therapy, since it is implicated in initiation, proliferation, and cancer cell survival. Curcumin has been found to inhibit the PI3K/Akt pathway in tumor cells, primarily via the regulation of different key mediators, including growth factors, protein kinases, and cytokines. This review presents the therapeutic potential of curcumin in different malignancies, such as glioblastoma, prostate and breast cancer, and head and neck cancers, through the targeting of the PI3K/Akt signaling pathway.

## 1. Introduction

Despite significant advances in treatment and a better understanding of the underlying principles of tumor development, cancer incidence is reckoned to be 28.4 million cases worldwide in 2040, compared to 19.3 million cases in 2020 [1,2]. In an attempt to improve the clinical outcomes of patients with cancer, different personalized care treatments have emerged. However, carcinogenesis is a multistep process where cellular and molecular alterations constantly make their appearance in all of its phases, including initiation, progression, and metastasis. Moreover, resistance to therapeutic options is due to a series of distinct mechanisms, including increased efflux of drugs, enhanced DNA repair mechanisms, epigenetic alterations, and a tumor-promoting microenvironment. Thus, the identification of novel, effective, and, most importantly, safe anti-cancer agents that can overcome drug resistance and improve the clinical outcomes of cancer patients is, nowadays, the primary objective in cancer treatment [3,4]. In this context, several studies that were both pre-clinical and clinical have focused on the investigation of the molecular mechanisms underlying the different stages of tumor development. 

Several molecular pathways have been widely studied for their implications in cancer progression and survival. Two key pathways that are frequently mutated and hyper-activated in tumors are the PI3K/Akt/mTOR signaling pathway and the Ras/Raf/MAPK pathway. Apart from separately promoting cancer cell proliferation, these two pathways are also strongly interconnected through different upstream and downstream regulators. Other pathways frequently altered in tumors include the p53-mediated apoptosis pathway, β-catenin/Wnt signaling pathway, and Notch signaling pathway [5].

The phosphatidylinositol-3-kinase (PI3K)/protein kinase B (AKT) signaling pathway is a key regulator of cell growth and the most activated pathway in different tumor types. Overactivation of this pathway drives cancer progression, survival, metastasis, and, ultimately, resistance to chemotherapy [6,7]. In specific, mutations in different components of the pathway, including the AKT oncogene, inactivation of the tumor suppressor gene PTEN, and the overexpression of growth factors—such as vascular endothelial growth factor-A (VEGF-A), platelet-derived growth factor (PDGF), or epidermal growth factor (EGF)—are primarily responsible for the dysregulation of the PI3K/Akt pathway [8,9]. In addition, there is significant crosstalk between the PI3K/Akt pathway and other commonly dysregulated signaling networks in cancer, including the Wnt/β-catenin pathway, as well as the RAF/MEK/ERK signaling pathway [10,11]. Thus, targeting the PI3K/Akt pathway is considered a promising therapeutic strategy in cancer treatment. 

**Figure 1:**
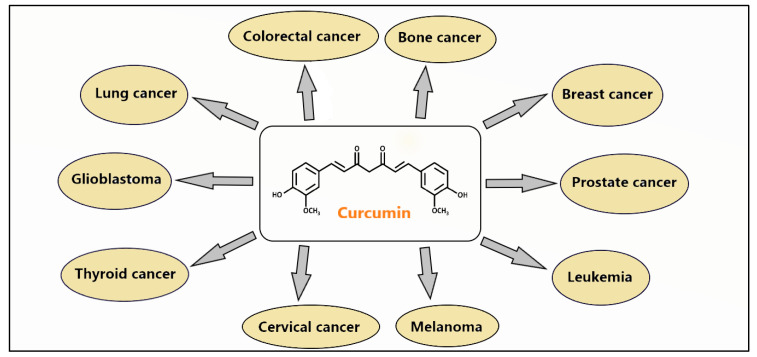
Examples of cancers against which curcumin has potential preventive and therapeutic effects.

Curcumin, also known as 1,7-Bis (4-hydroxy-3-methoxyphenyl)-1,6-heptadiene 3,5-dione, is a phenol-based component extracted from the rhizomes of Curcuma longa [12]. It belongs to a group of yellow-colored compounds known as curcuminoids. Curcumin has been found to possess a variety of biological properties, including its immunomodulatory, anti-inflammatory, antioxidant, neuroprotective, and anti-cancer effects. In addition, curcumin has been studied in different diseases, such as Alzheimer’s disease, pancreatitis, psoriasis, arthritis, cardiovascular diseases, and cancer. Curcumin has been reported to possess both preventive and therapeutic effects on several types of cancer, such as breast and prostate cancer, lung cancer, pancreatic malignancies, and brain tumors, including glioblastoma [3,4,5,6,7,8,9,10,11,12,13,14,15] (Figure 1). The anti-cancer properties of curcumin have been attributed to its diverse interactions with key molecular pathways implicated in different biological processes, such as proliferation, control of the cell cycle, apoptosis, metastasis, angiogenesis, and inflammation. Moreover, curcumin has been found to specifically target cancer stem cells, a population of cells largely responsible for the resistance to established chemotherapeutic treatments and recurrence of cancer patients. Furthermore, several studies have shown that curcumin may sensitize tumor cells to both chemo- and radiotherapy and, thus, improve treatment efficacy while reducing side effects when given as an adjuvant therapy [12,15]. This review focuses on the multifaceted role of curcumin in improving different malignancies, primarily through the regulation of the PI3K/Akt signaling pathway. 

## 2. Structure–Activity Relationships of Curcumin

Curcumin belongs to a group of bioactive compounds known as curcuminoids isolated from the roots of Curcuma longa. Curcuminoids make up to 3–8% of turmeric, and curcumin is the most abundant of the three, accounting for about 77% of total curcuminoid content. On the other hand, demethoxycurcumin accounts for about 17%, and bis-demethoxy curcumin accounts for 3–6% [16].

All three derivatives share a common basic structure, where two benzenemethoxy rings are joined by an unsaturated chain. The presence of a β-diketone group is important from a structural and biological point of view, since it enables curcuminoids to exist in a keto-enol form and allows all three compounds to form metal complexes (with metals including copper, mercury, and cadmium) and, thus, reduce metal-induced cytotoxicity. From a biological point of view, this structural property of curcumin has been widely studied in diseases such as arthritis and Alzheimer’s disease. Moreover, the presence of the diketone group, along with the two phenolic groups, allows curcumin to take part in different reactions in which electron transfer and hydrogen abstraction are involved. This is particularly useful in the case of reactions with free radicals. Curcumin’s reaction with reactive oxygen species (ROSs) results in the formation of curcumin–phenoxyl radicals that are more stable and less reactive than the initial free forms. Interestingly, the stabilization of superoxide radicals by curcumin has been considered equally efficient to the effects of key antioxidants, such as superoxide dismutase [17,18].

## 3. Pharmacokinetics of Curcumin and New Delivery Systems

Due to its variety of biological effects and implications in different diseases, curcumin has been described as “pharmacodynamically fierce”. On the contrary, its pharmacokinetic profile has been described as “feeble”, primarily due to its poor absorption, low bioavailability, and quick elimination. The chemical characteristics of curcumin that are mostly responsible for its unfavorable pharmacokinetic profile are its lipophilic nature, low water solubility, and low stability at physiological pH and under alkaline conditions. Moreover, curcumin is unstable in the presence of light, and quick photochemical degradation occurs when solid or soluble curcumin is exposed to light [18,19].

According to a previous study, 8 g of curcumin administrated orally in patients with advanced pancreatic cancer resulted in poor plasma levels peaking at 22–41 ng/mL [20]. Similar results were obtained when curcumin was administrated at doses of 440–2200 mg/day in patients with advanced colorectal cancer. Neither curcumin nor its active metabolites were detected in the plasma of those patients [21]. Studies of the pharmacokinetic profile of curcumin in healthy volunteers have also highlighted the compound’s poor bioavailability. In specific, after curcumin administration of a single oral dosage of 10–12 g in 12 healthy humans, the compound was not detected in their plasma, except for one volunteer [22].

In spite of its low oral bioavailability, curcumin may cross the blood–brain barrier thanks to its enhanced lipophilicity and low molecular weight. However, only a few studies in murine models have explored curcumin’s brain concentrations. Following oral administration, mice receiving 2.5–10 mg/day of curcumin for 4 months exhibited levels of curcumin reaching 0.5 µg/g of tissue [23]. On the contrary, mice injected with 100 mg/kg curcumin through intraperitoneal administration displayed brain concentration levels of 4–5 µg/g of tissue after 20–40 min [24].

To improve curcumin bioavailability, several strategies have been employed in the last decade, including novel drug delivery formulations (such as nanoparticles, liposomal curcumin, nanospheres, and cell-derived nanovesicles), the design of structural analogs of curcumin, and combinations of curcumin with adjuvant compounds, such as piperine [25]. Such strategies could overcome the unfavorable pharmacokinetic profile of curcumin and enhance its oral bioavailability, thus increasing its biological effects, including its anti-cancer potential. Table 1 summarizes the finalized clinical trials of curcumin in cancer patients in relation to pharmacokinetics, and Table 2 highlights novel curcumin formulations based primarily on the use of nanotechnology to increase curcumin’s anti-cancer effects in vivo.

## 4. PI3K/Akt Signaling and Cancer

**Figure 2:**
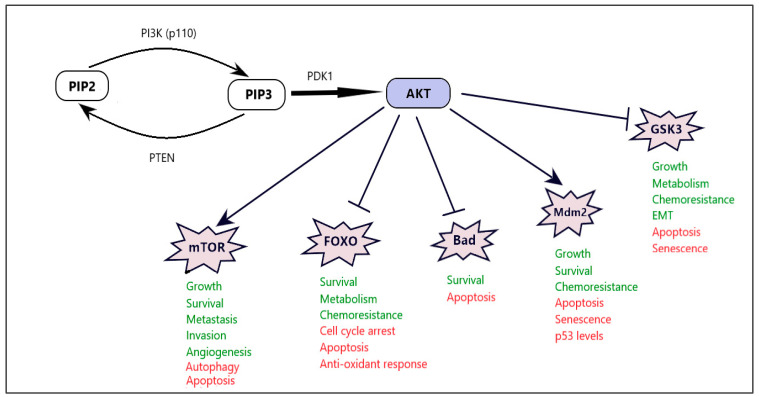
Downstream effectors of the PI3K/Akt pathway. Arrows (→) or blocking arrows (--|) show the effects of the activation of AKT on different effectors (stimulation or inhibition, respectively). Each downstream effector is implicated in different cellular processes, which are highlighted with green (induction) or with red (inhibition).

PI3K (class I) is a class of lipid kinases consisting of two units, a regulatory subunit (p85) and a catalytic subunit (p110) [38]. Following stimulation by external factors, including G-protein coupled receptors (such as the insulin receptor) or receptor tyrosine kinases (such as EGFR, VEGFR, and ERBB2), the p110 unit is activated and transforms phosphatidylinositol (4,5)-bisphosphate (PIP2) into the second messenger PIP3. This, in turn, causes protein kinase B (otherwise known as Akt) to translocate to the inner membrane, where it is then activated by the phosphoinositide-dependent protein kinase 1 (PDK1) [7]. There are three members of the Akt family, but only Akt1 is implicated in cancer development. Specifically, after activation, Akt1 phosphorylates and, thus, inhibits the activity of other downstream effectors (such as the mammalian target of rapamycin (mTOR) or Forkhead box O (FOXO), respectively), both of which play an important role in cell growth and survival. Other major effectors include the pro-apoptotic Bad protein, mouse double minute 2 (MDM2), which is an important regulator of the tumor suppressor p53, and GSK3, which is also implicated in several cellular processes, including tumor growth and metabolism (Figure 2). On the contrary, a phosphatase named PTEN converts PIP3 into PIP2, thus acting as a negative regulator of the PI3K/Akt pathway [39,40].

Members of the PI3K/Akt/mTOR pathway are commonly mutated in cancer. Somatic mutations in the PIK3CA gene that encodes the subunit p110 of class I PI3K, as well as PTEN mutations resulting in PTEN silencing, are the most prominent in cancer. PTEN, in particular, is implicated in several cellular processes, including cell growth, survival, and metabolism; thus, even small changes in its expression may result in major effects on cell function [41]. On the contrary, somatic AKT mutations have been found in about 5% of human cancer cases, including breast, ovarian, and colorectal cancer. However, AKT gene amplification, post-translational modifications, and dysregulation of its upstream effectors (including cytokine receptors and growth factors) also result in increased AKT-driven tumor development [42,43,44].

Apart from mutations and aberrations in the major nodes of the PI3K/Akt pathway resulting in its dysregulation in favor of cancer growth and survival, other factors, including non-coding RNAs, cell adhesion molecules, and oncogenes (such as RAS), may also interact with this pathway in different tumors. For example, miRNAs, such as mir-320 or mir-579, which are usually dysregulated in human cancers, may directly target major members of the PI3K signaling pathway, including PDK1, Akt, or mTOR [45,46]. Moreover, constitutively activated RAS signaling in tumors caused primarily by mutations in RAS or overexpression of RTKs has been linked to increased PI3K activation [47].

## 5. Curcumin and PI3K/Akt/mTOR Signaling

Curcumin has been widely studied as a promising anti-cancer agent for the treatment of different malignancies. Both in vitro and in vivo studies have shown that curcumin interacts with a variety of intracellular signaling pathways related to cancer development, survival, angiogenesis, and metastasis [48]. One such pathway is the PI3K/AKT/mTOR signaling pathway. Curcumin directly interacts with different molecules of this pathway, including the mammalian target of rapamycin complex 1 (mTORC1), AKT, PI3K, and growth factors, including EGFR [49,50,51]. In addition, curcumin indirectly targets the PI3K/Akt pathway through interaction with major upstream effectors, including IκΒ kinase B (IKKβ) and AMP-activated protein kinase (AMPK). In specific, curcumin has been found to inhibit IKKβ, an upstream regulator of mTORC1, resulting in the inhibition of tumor growth in breast and human adenoid cystic carcinoma cells. On the other hand, the upregulation of the AMPK, a kinase implicated in cell metabolism that, once activated, downregulates mTORC1, is another mechanism of the anticancer effect of curcumin in tumors, including prostate, breast, and lung cancer [52,53,54].

Accumulating evidence indicates that autophagy plays a complex role in cancer and is partly regulated by the PI3K/Akt/mTOR pathway. Studies have shown that the activation of mTORC1 reduces autophagy, whereas its inhibition results in the induction of autophagic flux. In fact, mTORC1 has been reported to directly phosphorylate the kinase complex responsible for the initiation of autophagy [55]. Curcumin can induce autophagy through the inhibition of the PI3K/AKT/mTOR axis, as has been previously shown in studies involving human lung cancer and glioblastoma cells [56,57].

The PI3K/Akt/mTOR pathway is also strongly related to apoptosis, the process of programmed cell death. Activation of this pathway results in the downregulation of pro-apoptotic proteins, particularly those of the bcl-2 family, including BAX, BAK, BAD, and BID. Notably, Akt inhibits apoptosis by directly phosphorylating BAD at Ser136, causing its release from the Bcl-xL complex. Furthermore, Akt activates the anti-apoptotic protein Bcl-2 to further promote cell survival [58,59,60]. Curcumin has been found to induce apoptosis in different cancers partly through the inactivation of the PI3K/Akt pathway. More specifically, curcumin can inhibit both Akt and mTOR phosphorylation [56,61] or upregulate the negative effector of the pathway, which is known as PTEN [50]. 

## 6. Curcumin against the PI3K/Akt Pathway in Different Malignancies

Curcumin exerts its anti-proliferative effects in different malignancies through interaction with the PI3K/Akt pathway. Table 3 highlights the anti-cancer effects of curcumin against different cancers mediated through the PI3K/AKT/mTOR pathway.

### 6.1. Glioblastoma

Malignant brain tumors are responsible for approximately 15,000 deaths in the US every year [82]. The most common and aggressive malignant brain tumor in adults is glioblastoma (GBM), with an incidence rate of about 3–4 people per 100,000 [83,84]. GBM treatment runs into a series of challenges, such as high levels of intratumoral and intertumoral heterogeneity, difficulty of delivery of chemotherapeutic agents across the blood–brain barrier, an immunosuppressive microenvironment, and, of course, several alterations in signaling pathways [85,86,87]. All of these challenges make the development of new therapeutic agents hard to achieve, and thus, in clinical practice, standard treatment is still based on the Stupp protocol. According to this, the maximally safe surgical resection is followed by a combination of chemotherapy and radiotherapy, with the only FDA-approved chemotherapeutic agent being temozolomide (TMZ). In the event of recurrence, which is almost always the case in GBM patients, different systemic therapy options have evolved, including bevacizumab, nitrosoureas, immunotherapy, oncolytic virus therapy, or CAR T cell therapy. However, all available therapeutic options fail to significantly increase GBM patients’ survival and are usually accompanied by severe adverse effects. Thus, the need to identify novel molecules with potent anti-glioma effects when given alone or in combination with standard therapy is pivotal. In that direction, it is important to focus on the molecular pathways that are commonly dysregulated in most GBMs [83,88].

One of the most altered pathways in GBM is the PI3K/Akt/mTOR signaling pathway. It has been estimated that this pathway is aberrantly activated in almost 90% of GBM cases and acts as a pro-survival network, availing tumor growth, survival, and resistance to therapy [89,90]. Moreover, patients with an over-activated PI3K/Akt pathway have a poor prognosis and survival compared to patients without activation of this pathway. The most common alteration in the PI3K/Akt axis in GBM has been related to the loss of PTEN function due to mutations, such as point mutations in its coding region or deletions [91,92]. Moreover, according to data obtained from the Cancer Genome Atlas pilot project, *PI3K* mutations, as well as *AKT* amplification, have also been observed in GBM samples [93]. However, the overexpression of EGFR, as well as the loss of function of the tumor-suppressor PTEN, is the most prominent reason for the overactivation of PI3K or Akt in GBM [94].

**Figure 3:**
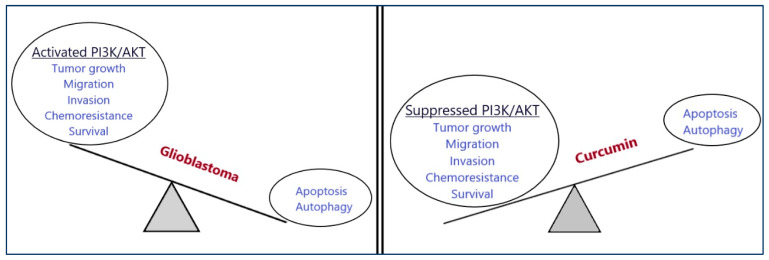
Anti-tumor effect of curcumin on human glioblastoma through the downregulation of the PI3K/Akt pathway.

Curcumin has been found to regulate important signaling pathways in GBM, including the PI3K/Akt signaling pathway (Figure 3). In a study by Zanotto-Filho et al., curcumin inhibited GBM progression primarily by targeting the PI3K/Akt pathway in the human GBM cell lines U87, U373, and U138MG. The exact mechanism of action included the inhibition of the phosphorylation of AKT at Ser473. Interestingly, the cytotoxic effects of curcumin were irrespective of the PTEN or p53 mutational status of GBM cell lines [68]. In another study by Wang et al., curcumin significantly decreased the levels of p-AKT and p-mTOR in human GBM U251 and U87 cells, resulting in the suppression of GBM proliferation and migration. Furthermore, curcumin promoted apoptosis in the same study through the induction of PTEN and p53 protein expression [69].

Apart from the induction of apoptosis in human GBM cells, curcumin has also been studied for its effects on other types of cell death, including autophagy and paraptosis in relation to PI3K/Akt/mTOR signaling. Generally, the role of autophagy in human cancer is complicated, and an intercommunication seems to exist between autophagy and apoptosis. In specific, autophagy has been found to either induce an alternative-to-apoptosis “suicidal” type of cell death that leads cancer cells to extinction under extensive damage or play a protective role in the initial stages of tumor formation. In this context, potent agents that inhibit autophagy at an early phase of tumor formation have been developed, and so have potent inducers of autophagy [95]. Autophagic cell death in GBM is a complex process of degradation of cellular components that can affect the growth, survival, and migration abilities of GBM cells [96]. The relationship between autophagy and the PI3K/Akt axis has been established, and the latter seems to inhibit autophagic cell death in GBM by negatively regulating the activity of autophagy-related proteins [97]. This is why several autophagy inducers targeting the PI3K/AKT/mTOR pathway have been synthesized or studied for their anti-cancer effects against different tumors, including GBM. In this context, curcumin has been evaluated for its ability to induce autophagic cell death in human GBM cells. According to Aoki et al. and Maiti et al., curcumin induced non-apoptotic autophagic cell death in human GBM cell lines U87 and U373 through the inhibition of the PI3K/Akt pathway and its downstream effector p70S6K. In specific, curcumin downregulated the levels of p-AKT, total AKT, p-mTOR, and PI3Kp85, as well as the phosphorylated form, suggesting that it promotes mTOR-dependent autophagic cell death in these GBM cell lines [57,98]. Aoki et al. also showed that the addition of recombinant human AKT1 protein weakened autophagy-induced cell death caused by curcumin [98]. In another study by Zhang et al., curcumin reduced both cell migration and invasion of A172 GBM cells through an mTOR-dependent induction of autophagy [99].

The tumorigenic features of glioblastoma stem cells (GSCs) are partly responsible for the aggressiveness and invasiveness of GBM. Overactivation of the PI3K/Akt/mTOR signaling pathway has been strongly related to the sustaining of the invasive phenotype of this cell population [100]. Curcumin has been reported to repress the invasiveness of GSCs through the downregulation of the PI3K/Akt signaling pathway [101]. Moreover, when highly invasive A1207 and SNB19 human GBM cell lines were treated with curcumin, their migratory ability was impeded due to the downregulation of the oncogenic NEDD4 protein. Since NEDD4 induces PTEN degradation and, thus, the activation of the PI3K/Akt axis, it is shown that curcumin inhibits the migration and invasion of GBM cells in an AKT-dependent way [70]. 

Lastly, it is worth mentioning that curcumin has also been studied for its ability to induce AKT- and p53-dependent paraptosis in human GBM cells through the regulation of different micro-RNAs [102]. Paraptosis is a type of cell death characterized by cytoplasmic vacuolization and mitochondrial damage [103]. When A172 cells were treated with curcumin, morphological changes characteristic of this alternative way of cell death were observed because of the downregulation of both the AKT and p53 pathways. The effects of curcumin on these two signaling axes were attributed to the regulation of miRs, such as miR-223-3p, miR-21-5p, and miR-151-3p. Most importantly, the downregulation of the expression of miR-27a was the eminent reason for the induction of paraptosis by altering the integrity of the reticulum [102].

### 6.2. Lung Cancer

Despite advances in treatment options, lung cancer remains a leading cause of cancer death worldwide. Small-cell lung cancer (SCLC) and non-small-cell lung cancer (NSCLC) are the two major subtypes of lung cancer, with NSCLC accounting for about 85% of patient cases [104]. In clinical practice, surgical resection, chemotherapy, and radiotherapy are the primary treatment options for the early stages of NSCLC. On the contrary, stages III and IV are treated with chemotherapy and radiotherapy alone. Due to the significant limitations of all traditional chemotherapeutic drugs, including non-specific targeting and damage to normal tissues, molecular targeted therapy for the treatment of lung cancer has considerably evolved. The major targets of this therapeutic option are receptor tyrosine kinases, with the most prominent being EGFR. However, vast tumor heterogeneity and acquired drug resistance limit the efficacy of targeted therapy as well. In this context, the identification of agents that can specifically target molecular pathways that are frequently dysregulated in lung cancer is crucial for improving the clinical outcomes of patients [105,106]. Alterations in different components of the PI3K/Akt/mTOR pathway, including somatic mutations of PIK3CA, deletions of PTEN, and increased Akt activity, have been recorded in most NSCLC cases, resulting in this pathway being one of the most frequently deregulated in lung cancer [107,108,109]. 

Curcumin has been found to both prevent and treat NSCLC by acting on different aspects of tumor development, metastasis, invasion, and cell death [56]. According to Liu et al. and Wang et al., curcumin inhibits the proliferation of NSCLC A549 and H1299 cells through the downregulation of Akt and mTOR, resulting in the induction of two different cell death mechanisms, apoptosis, and autophagy [56,110]. In another study by Wang et al., the anti-metastatic and anti-invasive effects of curcumin on NSCLC cells were investigated in relation to miR-206. The latter has been found to be downregulated in different tumors, including lung cancer, resulting in the upregulation of major molecules of the PI3K/Akt axis. Curcumin was found to enhance the expression of miR-206 and, thus, inhibit the phosphorylation of both Akt and mTOR [74]. The effect of curcumin on the expression levels of other important microRNAs has also been investigated by Jin et al., and their results suggested that curcumin promotes apoptosis in NSCLC cells via the upregulation of miR-192-5p, resulting in the suppression of the PI3K/Akt axis [75]. In another study, the cytotoxic effects of curcumin against A549 cells were attributed to its ability to increase PTEN expression through the suppression of miR-21 [111].

The interaction among curcumin, 14-3-3 proteins, and the PI3K/Akt signaling pathway has also been investigated in A549 lung cancer cells; 14-3-3 proteins are important regulators of Bcl-2 family members, including Bad, Bax, and Bid, and they are often upregulated in different cancer cell lines. Moreover, 14-3-3 proteins may promote cell growth and survival by activating Akt by binding to the p85 subunit of PI3K [112,113]. Curcumin has been found to induce Bad-mediated apoptotic cell death in A549 cells through the suppression of both 14-3-3 and Akt levels [76]. 

In terms of angiogenesis and the epithelial–mesenchymal transition (EMT), both of which are critical events in lung cancer progression, curcumin has been found to inhibit both events through the suppression of the PI3K/Akt/mTOR pathway. In specific, when two human lung cancer cell lines, A549 and PC-9, were treated with curcumin, a dose-dependent decrease in the expression of Akt and mTOR was observed as a result of the inhibition of hepatocyte growth factor (HGF) and its downstream effector c-Met [112]. Activation of the HGF/c-Met signaling pathway increases invasion, metastasis, and angiogenesis primarily through the upregulation of other important pathways, including the PI3K/Akt and the JAK/STAT pathways. Thus, curcumin may inhibit HGF-induced angiogenesis and invasion in a PI3K/Akt-dependent cascade [114,115].

### 6.3. Breast Cancer

Breast cancer remains the most common type of malignancy among women today. It is a highly heterogeneous disease and is further divided into different subtypes depending on the expression of hormone receptors and human epidermal growth factor 2 (ERBB2, also known as HER2) [116]. In specific, there are three major subtypes of breast cancer: hormone-receptor-positive/ERBB2-negative, ERBB2-positive, and, lastly, triple-negative breast cancer. The majority of patients (about 70%) fall into the first category. Most breast cancers are not metastatic at early diagnosis, and the preferred treatment plan includes local therapy, which is either breast-conserving therapy (lumpectomy followed by radiotherapy) or mastectomy. Systemic treatment for hormone-receptor-positive tumors includes endocrine therapy with the well-established agent tamoxifen, and in high-risk patients, the other chemotherapeutic agents used in clinical practice include doxorubicin, cyclophosphamide, and paclitaxel [117,118].

On a molecular level, one of the most dysregulated signaling pathways in breast cancer is the PI3K/Akt/mTOR pathway. It has been estimated that about 30% of patients with breast cancer have PIK3CA mutations, followed by PTEN loss due to somatic mutations or epigenetic alterations [119]. The importance of targeting the PI3K/Akt pathway in breast cancer has led to the investigation of potential PI3K and mTOR inhibitors to act synergistically with chemotherapy or hormone therapy [120]. 

Curcumin has been found to exert its antiproliferative effects in different breast cancer cell lines. According to Jia et al., there is a difference in response to curcumin between hormone-dependent MCF-7 and hormone-independent MDA-MB-231 cells. In the MDA-MB-231 cell line, curcumin hindered the phosphorylation of Akt in both a time- and dose-dependent way. On the contrary, in MCF-7 cells, the effect of curcumin on the phosphorylation status of Akt was more complicated, since at lower curcumin concentrations, there was an increase in the activation of Akt, whereas at higher concentrations, curcumin treatment resulted in a decrease in Akt phosphorylation. Interestingly, when both cell lines were co-treated with curcumin and an Akt inhibitor, curcumin-resistant MCF-7 cells became more susceptible to curcumin [121]. In a similar context, Kizhakkayil et al. observed that co-treatment with curcumin and the PI3K inhibitor LY294002 induced apoptosis in MCF-7 breast cancer cells through the attenuation of the curcumin-induced phosphorylation of Akt and activation of GSK3β [62]. The mechanism underlying the cytotoxic effects of curcumin on several breast cancer cell lines in relation to PI3K/Akt signaling has been investigated by Hu et al. Their team used seven different cell lines, and the results showed a differential susceptibility to curcumin treatment between ER+ breast cancer cells, including MCF-7, T4FD, MDA-MB-415, and ER-PR-HER2 cells, such as MDA-MB-231 and BT-20. In specific, ER+ cells were more sensitive to curcumin treatment. In the same study, curcumin was found to induce the cell cycle and apoptosis through the inhibition of both Akt and mTOR phosphorylation and, consequently, the inhibition of their downstream effectors, such as protein S6 [63].

The effect of curcumin on HER-2 overexpressed breast cancer cell lines, specifically, BT-474 human ductal breast cancer cells and SKBR3, was evaluated by Lai et al. According to their results, the downregulation of HER-2 and EGFR in curcumin-treated cells was strongly related to the inhibition of signaling pathways, including PI3K/Akt (in which a decrease in the phosphorylation status of Akt was observed after curcumin treatment), MAPK, and NF-κΒ [64]. 

Another aspect of the anticancer potential of curcumin in human breast cancer has to do with its effects on the expression of microRNAs, with the most prominent being miR-21. In a recent study by Wang et al., curcumin reduced the expression of miR-21 in MCF-7 breast cancer cells dose-dependently. Moreover, curcumin inhibited the phosphorylation of Akt and increased the expression of PTEN. Since it has been established that there is a negative association between miR-21 and PTEN, the anticancer effects of curcumin in this study may be attributed to the miR-21/Akt/PTEN molecular signaling pathway [65,122]. The relationship among curcumin, microRNAs, and the PI3K/Akt axis in MCF-7 breast cancer cells has also been explored by Li et al. In their study, curcumin reduced the expression of the oncogenic miR-19, increased the expression of PTEN, and decreased the phosphorylation of Akt. In that way, curcumin managed to protect cells from the tumor-promoting effect of Bisphenol A, a well-established endocrine disrupter [123].

### 6.4. Prostate Cancer

According to the Global Cancer Observatory, there were 375,304 deaths from prostate cancer in 2020 worldwide [124]. This type of cancer mostly affects middle-aged men and presents a heterogeneous disease, mainly on the basis of genetics. Typically, prostate cancer can be divided into two major subtypes depending on the sensitivity to androgen receptor signaling (androgen-sensitive or -insensitive). Available conventional therapeutic options include radical prostatectomy, chemotherapy, and androgen deprivation therapy. Moreover, precision medicine for men with advanced prostate cancer has also emerged as an alternative gene-specific treatment modality. However, all available treatment options against prostate cancer relate to severe side effects that limit their efficacy, and on top of that, patients frequently develop resistance to these treatments [125]. In this context, research has now focused on targeted therapy based on dysregulated cellular pathways and the exploitation of medicinal plants and their compounds in that direction. Several studies have examined the role of PI3K/Akt signaling in the development and progression of prostate cancer. The results show that abnormal expression of major components of this pathway is a common event in prostate cancer patients [126]. Hyperactivation of the PI3K/Akt/mTOR signaling pathway in prostate cancer is mostly related to mutations in PTEN; however, other abnormalities, such as gain-of-function mutations in PIK3CA or AKT genetic aberrations, have also been detected [127,128]. 

Curcumin has been found to promote cell cycle arrest and apoptosis in both androgen-dependent and androgen-independent prostate cancer cells in vitro through the downregulation of the PI3K/Akt pathway. Specifically, treatment of LnCaP and C4-2B cells with curcumin resulted in a PTEN-modulated induction of apoptosis and cell cycle arrest in both cell lines, suggesting that the effects of curcumin are independent of the androgen-dependent/-independent status of the cells [129]. In another study by Shankar et al., curcumin reduced viability and induced apoptosis in three prostate cancer cell lines, LnCaP, PC-3, and DU145, via the regulation of two pivotal components of the PI3K/Akt/mTOR pathway, PI3K and Akt. Interestingly, curcumin inhibited the expression of both subunits of PI3K (p85 and p110), as well as the phosphorylation of Akt at Ser 473. In the same study, treatment of cells with PI3K inhibitors, such as LY294002, increased the apoptotic potential of curcumin [77]. 

In a similar context, Yu et al. treated PC-3 cells with curcumin and found that a dose- and time-dependent inhibition of phosphorylation of Akt and mTOR occurred, resulting in the downregulation of their downstream effectors as well. On the other hand, treatment of the cells with Calyculin A, a phosphatase inhibitor with the ability to phosphorylate major compounds of the PI3K/Akt pathway at serine/threonine, prior to curcumin treatment reversed the inhibitory effects of the latter, suggesting that curcumin’s antiproliferative effects are regulated through a phosphatase-dependent mechanism [78].

### 6.5. Colorectal Cancer

Colorectal cancer remains a challenge nowadays, accounting for about 9.4% of total cancer death cases [2]. It is a multi-factorial disease, and, in most patients, different molecular pathways are co-dysregulated [130]. For early-stage colorectal cancer patients, endoscopic treatment remains the gold standard in clinical practice. On the other hand, for metastatic colorectal cancer treatment, surgery coupled with chemotherapy or radiotherapy is the most common therapeutic scheme. Moreover, over the last few years, special attention has been given to the use of combinations of chemotherapeutic agents, such as trifluridine/tipiracil hydrochloride, in an attempt to improve the clinical outcomes of patients. Molecular-targeted drugs and immune checkpoint inhibitors have also been approved for the treatment of colorectal cancer. However, common challenges related to this type of cancer that limit the efficacy of current treatment options include drug resistance and high recurrence rates [131]. In that respect, identifying molecular pathways that are frequently dysregulated in colorectal cancer and relate to tumor progression and survival is crucial. One such pathway is the PI3K/Akt signaling pathway. In colorectal cancer, the most commonly found alterations of this pathway include PIK3CA mutations and loss of PTEN activity [132]. These changes result in the hyperactivation of the PI3K/Akt/mTOR pathway and are partly responsible for the metastatic initiation events of colorectal cancer cells, as well as the gaining of chemoresistance [133]. Thus, targeting this pathway has garnered the interest of several scientific groups.

Curcumin has been found to inhibit PI3K/Akt signaling in LoVo human colon cancer cells primarily through the downregulation of p-Akt levels. This, in turn, results in an increase in apoptosis-related molecules, including Bax and caspace-3 [66]. The role of curcumin in an acquired 5-Fu-resistant LoVo cell line was also investigated by Ma et al. Curcumin treatment partly warded off the resistance of cancer cells to the chemotherapeutic agent 5-Fluoouracil through the regulation of the PI3K/Akt pathway. In specific, curcumin downregulated the phosphorylation of PI3K, Akt, and mTOR, resulting in a decrease in the EMT of colon cancer cells [134].

In another study by Chen et al., the cytotoxic effects of curcumin on PTEN-deficient human colorectal cancer cells were evaluated in comparison with HCT116 PTEN+ cells. Although curcumin decreased cell viability in both cell lines, its cytotoxic effects were more prominent in PTEN-deficient cells. The researchers proposed that PTEN deficiency led to the alteration of the curcumin-induced cell cycle arrest pattern in relation to the PTEN-regulated PI3K/Akt/p21 molecular pathway [135]. The effect of curcumin on mTOR, an important downstream effector of the PI3K/Akt signaling pathway, has been investigated by Johnson et al. According to their results, curcumin suppressed the expression of both mTORC1 and mTORC2 in HCT116 colorectal cancer cells. In the same study, curcumin treatment also resulted in an increase in Akt phosphorylation at Ser 473; however, this may be attributed to the downregulation of PHLPP1, a key inhibitor of Akt [67].

### 6.6. Thyroid Cancer

Thyroid cancer is a common endocrine malignancy representing 1–4% of all malignancies. Among the different histopathological types, papillary thyroid cancer (PTC) accounts for about 85–95% of new thyroid cancer cases [136]. In many cases, PTCs are recognized as either papillary microcarcinoma (PMC) or follicular thyroid microcarcinoma, and in both cases, thyroid nodule sizes are less than 1 cm in diameter; thus, active surveillance is the primary strategy. Since most PMCs remain stable for a long period after the first measurement, surgery is not always required. However, in cases where the nodule size increases by at least 3 mm from the initial diagnosis or when symptoms of invasion of the trachea appear, the transition to surgery is likely to occur. Moreover, radioactive iodine therapy is usually recommended for high-risk patients. In recent years, targeted therapies have also evolved from the study of the genetic landscape surrounding thyroid cancer, resulting in the approval of two tyrosine kinase inhibitors by the FDA. In view of this, studying the molecular pathways involved in thyroid cancer progression may hold a bright future in improving the therapeutic options of thyroid cancer patients [137,138]. Thyroid malignancies are characterized by the disruption of different molecular pathways, including the PI3K/Akt pathway. In specific, PIK3CA and PTEN are the two most frequently mutated genes in thyroid cancer. These mutations may result in the continuous activation of major downstream effectors of the pathway, such as mTOR. This, in turn, promotes tumorigenesis and metastasis [139,140].

Curcumin has been found to exhibit its anti-tumor effects on FTC133 human thyroid cancer cells primarily through the inhibition of the PI3K/Akt signaling pathway. In a study by Xu et al., curcumin inhibited the phosphorylation of both PI3K and Akt, resulting in the suppression of cell viability, metastasis, and invasion of FTC133 cells [79]. Using the same cell line, Zhang et al. showed that curcumin treatment for 14 days resulted in a long-term suppression of cell viability. The inhibitory effect of curcumin on cell colony formation was determined as well. Moreover, co-treatment of FTC133 cells with curcumin and an approved kinase inhibitor known as sorafenib resulted in a synergistic inhibition of cell viability through the regulation of the PI3K/Akt and ERK signaling pathways [141]. 

In another study by Zhang et al., the role of curcumin in the induction of autophagy was investigated in a series of thyroid carcinoma cell lines, including FTC133, BCPAP, and the 8505C anaplastic thyroid cancer cells. Curcumin was found to induce autophagy at various concentrations partly through the downregulation of the PI3K/Akt/mTOR pathway. In specific, after curcumin treatment, the levels of phosphorylated Akt, PDK1, protein S6, and p70S6K were significantly reduced. On the contrary, curcumin did not promote autophagic cell death in normal thyroid epithelial cells [80]. Inhibition of the PI3K/Akt/mTOR pathway by curcumin has also been related to the restoration of sensitivity to radioiodine of dedifferentiated thyroid cancer cells. Curcumin can regulate the expression of important thyroid-specific proteins, including the sodium/iodide symporter (NIS) and, thus, improve the therapeutic effect of radioiodine [81].

### 6.7. Leukemia

Leukemia is an aggressive malignancy of the blood that can be categorized into four major subgroups: acute myeloid leukemia (AML), chronic myeloid leukemia (CML), acute lymphoblastic leukemia (ALL), and chronic lymphocytic leukemia (CLL). In 2020, leukemia was responsible for about 3% of all cancer death cases globally [142]. The management of leukemia varies significantly, depending primarily on the specific subtype, as well as patient-related factors.

ALL is characterized by genetic alterations related to both the differentiation and development of lymphoid precursor cells [143]. The presence of the Philadelphia chromosome is the key molecular indicator of the therapeutic strategy clinicians that are entitled to follow. For Ph-positive ALL patients, tyrosine kinase inhibitors (TKIs) are the primary therapeutic choice, usually in combination with chemotherapeutic agents. On the other hand, TKIs are not advantageous in Ph-negative patients. In many cases, Ph-positive ALL patients show poor clinical outcomes, primarily due to the molecular and genetic heterogeneity of this disease entity [144]. The PI3K/Akt pathway seems to play a significant role in this type of malignancy. Mutations in PIK3CA and PIKRA, encoding the p110a and p85a subunits of PI3K, respectively, have been observed in both T-cell ALL and B-cell ALL. Moreover, mutations in exon 2 of AKT1 have also been reported. Since PI3K/Akt activation is important for the adhesion of cancer cells to bone marrow stromal cells, targeting this pathway has gained the attention of several researchers [145,146]. In B-precursor ALL cells, curcumin was found to downregulate PI3K/Akt signaling, primarily through the dephosphorylation of the continuously activated AKT/PKB. This resulted in the induction of apoptosis through the inhibition of the anti-apoptotic bcl-2 protein, along with an upregulation of the pro-apoptotic Bax [61]. Similar results were obtained by Hussain et al., who investigated curcumin for its cytotoxic effects on T-cell ALL. According to this scientific group, curcumin treatment resulted in the dephosphorylation of AKT and, consequently, the inactivation of its downstream effectors FOXO and GSK3 [71].

The potential anti-tumor effect of curcumin when given in combination with approved chemotherapeutic agents in relation to PI3K/Akt signaling has also been investigated by different scientific groups. Wang et al. co-treated ALL cells with curcumin and L-asparaginase. Their results suggest that curcumin potentiates the cytotoxic effects of L-asparaginase through the dephosphorylation of AKT and its related proteins, such as FOXO1 and GSK3β [147]. Guo et al. investigated the anti-leukemia effects of curcumin alone and in combination with imatinib in Ph+ ALL cells. According to their results, curcumin mediated apoptotic cell death partly through the inhibition of PI3K/Akt signaling. Moreover, it effectively inhibited the overactivation of the PI3K/Akt/mTOR pathway that has been observed because of imatinib treatment and, thus, sensitized cancer cells to the cytotoxic effects of the latter [72].

CLL accounts for most adult leukemia cases in the Western world. Treatment options for CLL vary significantly and highly depend on the clinical profile and symptoms of patients. When required, the initial treatment may involve chemotherapy, targeted therapy, monoclonal antibodies, or different combinations of the above. The implication of the PI3K/Akt signaling pathway in this malignancy has been evinced by the development and approval of a series of PI3K inhibitors, including idelalisib and duvelisib [148]. Curcumin has been investigated as a potential anti-leukemic agent against B-cell CLL. In a study by Ghosh et al., curcumin was found to inhibit Akt phosphorylation and overcome stromal protection of CLL cells at higher doses [149]. In another recent study that involved TP53-mutated CLL cells, curcumin was also found to inhibit cell proliferation and act synergistically with ibrutinib through the downregulation of the PI3K/Akt pathway [150].

AML is an aggressive bone marrow malignancy that is characterized by the dysregulation of the PI3K/Akt/mTOR axis in about 60% of patients. A series of mutations, gene amplifications, and deletions play a key role in the constitutive activation of this pathway, resulting in the survival of cancer cells and resistance to chemotherapy [151]. Curcumin has been investigated as a potential cytotoxic compound against a series of AML cell lines, including HL-60, ML-2, and U937, by Zhou et al. The results showed that curcumin primarily targeted Akt in all tested cell lines, but the sensitivity of different AML cells to curcumin was not fully correlated with the Akt phosphorylation status, suggesting that there may be crosstalk between PI3K/Akt and other pathways affected by curcumin [73]. Wu et al. also found that a major metabolite of curcumin named tetrahydrocurcumin sparked autophagic cell death in AML cells through the downregulation of the PI3K/Akt pathway [152].

## 7. Conclusions and Future Perspectives

Curcumin, a polyphenol extracted from C. longa, is a natural compound with promising biological properties, including its anti-cancer effects. It has been found to regulate different signaling pathways implicated in cancer development, metastasis, and resistance to therapy. As discussed, curcumin can suppress the PI3K/Akt/mTOR signaling pathway and its downstream effectors. Since this pathway is overactivated in most tumors and relates to tumorigenesis and cancer progression, suppressing it may pose a promising therapeutic strategy. Moreover, in order to overcome the limits of current chemotherapeutic treatments with the aim of improving patients’ clinical outcomes, research should focus on the utilization of curcumin and its analogs in combination with existing approved drugs. 

Several in vitro and in vivo studies have highlighted the potential of curcumin as an anti-cancer agent. Nevertheless, clinical studies are limited, primarily due to the unfavorable pharmacokinetic profile of the compound. The incorporation of novel technologies, such as nanotechnology, is crucial for the development of novel curcumin delivery formulations that can address the current bioavailability issues. Moreover, the development of curcumin analogs with a more favorable pharmacokinetic profile could enhance treatment efficacy and offer benefits, such as selective accumulation in cancer tissues and the slowdown of curcumin metabolism. In the same direction lies the development of novel curcumin mimics with partial structural resemblance to curcumin and added pharmacophores that may target specific cancer-related molecules, such as proteins or receptors that are highly expressed in tumors. Further clinical research is required to evaluate both the efficacy and safety of highly bioavailable curcumin in a clinical environment. This will shed new light on the remarkable pharmacological profile of curcumin for the management of different tumors. As more clinical evidence becomes available, curcumin is likely to become a fundamental part of cancer therapies in the future. 

## Figures and Tables

**Table 1:** Examples of finalized clinical trials on curcumin’s bioavailability in cancer patients.

Cancer	No. of Patients	Formulation, Dosage, and Delivery Scheme	Results	Ref.
Pancreatic or biliary tract cancer	16	- Curcumin dispersed with colloidal nano-particles (Theracurcmin) given orally. - Level 1: 200 mg, Level 2: 400 mg	Peak plasma curcumin levels 2 h after Theracurmin administration were 324 ng/mL at level 1 and 440 ng/mL at level 2	[26]
Colorectal cancer	12	- Capsules containing 450 mg of curcumin, 30 mg of demethoxy curcumin, and 20 mg of bis-demethoxy curcumin - 3 dose levels (1, 4, or 8 capsules per day) for 7 days before surgery	Tissue levels of curcumin 6 h after the last dosage were 0.9 ± 0.4, 6.7 ± 1.6, and 7.7 ± 1.8 for dose levels 1, 2, and 3, respectively	[27]
Hepatic metastatic disease from primary colorectal cancer	12	- Capsules containing 450 mg of curcumin, 30 mg of demethoxy curcumin, and 20 mg of bis-demethoxy curcumin - 3 dose levels (1, 4, or 8 capsules per day) for 7 days	No quantifiable levels of curcumin in plasma of patients	[28]
Adenocarcinoma of the colon or rectum	15	- Soft gelatin capsules containing 20 mg of curcuminoids (18 mg curcumin and 2 mg demethoxy curcumin) - 2, 4, 6, 8, or 10 capsules/per day containing 36, 72, 108, 144, or 180 mg of curcumin for up to 4 months	- No curcumin or its metabolites detected in plasma or urine at up to 29 days after first administration - Levels of curcumin in fecal samples at day 29 were 144–519 nmol/g for dosage of 144 mg curcumin and 64–1054 nmol/g for 180 mg of curcumin	[21]
Adenocarcinoma of the colon or rectum	15	- Capsules containing 500 mg of curcuminoids (450 mg of curcumin, 40 mg of demethoxy curcumin, and 10 mg of bis-demethoxy curcumin) - 1, 2, 4, or 8 capsules daily	- Plasma curcumin levels on days 2, 8, and 29 and at 1 h post administration of 8 capsules were 11.1+/− 0.6 nmol/L - Urine curcumin levels at the highest dosage were 0.1–1.3 nmol/L free curcumin, 19–45 nmol/L curcumin sulfate, 210–510 nmol/L curcumin glucoronide	[29]
Pancreatic cancer	25	- Capsule containing 1 g of curcuminoids (900 mg of curcumin, 80 mg of demethoxy curcumin, and 20 mg of bis-demethoxy curcumin) - 1 capsule daily orally for 8 weeks	- Conjugated plasma curcumin levels were 22–41 ng/mL at day 3, no detectable free or unconjugated curcumin	[20]

**Table 2:** Examples of various strategies for enhancing curcumin’s bioavailability in animal models of cancers.

Strategy	Curcumin Concentration	Delivery Method	In Vivo Anti-Cancer Results	% Success	Ref.
Dendrosomal curcumin	12.5 mg/kg body weight daily for 3 weeks	Intraperitoneal injection	Reduction in tumor size of BALB/c tumor-bearing mice, increase in survival	- The curcumin-treated tumor-bearing mouse group showed reduction in tumor volume at day 21 - All treated tumor-bearing mice were still alive by day 70	[30]
Curcumin-loaded PLGA nanospheres	25 μg PLGA-CUR NPs once	Intratumor injection	Inhibition of tumor growth in a C4-2 xenograft mouse model	The treated group of mice with C4-2 xenograft tumors showed about 17% tumor growth compared to 38% in the control group	[31]
Curcumin–chitosan nanoparticles	190 mg/kg for 4 weeks (3 times weekly)	Intravenous injection	Inhibition of tumor growth and angiogenesis in HepG2 xenograft mice	The treated group of mice showed a decrease in tumor volume 24 days after the first injection (407.5 mm^3^ for curcumin-PBCA nanoparticles vs. 936 mm^3^ for physiological saline)	[32]
β-cyclodextrin-curcumin complex	100 mg/kg in 10 mL of 0.9% sodium chloride daily for 14 days	Oral administration	Reductions in tumor volume and growth rate of tumors in a mouse hepatoma H22 tumor model	The treated group of tumor-bearing mice showed a 34.64% tumor inhibition rate compared to 0% in the control group	[33]
Curcumin nanogel conjugate	6 mg/kg twice every week	Intraperitoneal injection	Inhibition of tumor growth in a MiaPaCa-2 xenograft murine model	The treated group of mice showed a 15-fold decrease in the mean tumor volume on Day 49 compared to the control group	[34]
Liposomal curcumin (LC)	15, 30, 50, or 80 µM of LC for 6 h	Oral administration (incubation with LC)	Suppression of tumor growth in a zebrafish transplantation tumor model of endometrial cancer	The LC-treated group of zebrafish showed decrease in tumor volume by around 2-fold compared to the control 3 days after treatment at 50 μΜ LC	[35]
Liposomal co-delivery of curcumin and piperine	50 mg/kg once per day for 20 days	Intraperitoneal injection	Tumor growth inhibition and reduction in tumor volume in subcutaneous A549 tumor-bearing mice	The treated group of tumor-bearing mice showed a 41.04% tumor inhibition rate compared to the control group	[36]
Curcumin in bovine-milk-derived exosomes	20 mg/kg curcumin and 80 mg/kg exosomes 3 times per week	Oral gavage	Tumor growth inhibition in human cervical cancer (CaSki) xenografts in nude mice	Treated group showed greater inhibition of tumor growth (61%) compared to that of EXOs alone (21%)	[37]

**Table 3:** Examples of PI3K-/Akt-mediated effects of curcumin on cancer cell lines.

Cancer Type	Curcumin Treatment	Cell Line	Results and Mechanism	Year	Ref
Breast	10, 20, 30, 40 μΜ curcumin for 3, 6, 12, and 24 h	MCF-7 MDA-MB-231	Treatment with curcumin decreased AKT phosphorylation dose- and time-dependently	2014	[62]
	10 or 30 μΜ curcumin for 12 h	T47D, MCF7, MDA-MB-415, SK-BR-3, MDA-MB-231, MDA-MB-468, BT-20	Treatment with curcumin inhibited the phosphorylation of Akt, mTOR, and S6 after 12 h of treatment.	2018	[63]
	1–25 μg/mL (=2.7–67.9 μΜ) curcumin	MCF-7, MCF-10A, MDA-MB-231, BT-474, SK-BR-3-hr	Treatment with curcumin at high concentrations decreased the expression of p-AKT in BT-474 and SK-BR-3-hr	2012	[64]
	0, 1, 2, and 5 µM curcumin for 48 h.	MCF-7	Treatment with curcumin decreased p-AKT levels and increased PTEN levels dose-dependently	2017	[65]
Colorectal	40, 80, and 122 μΜ curcumin for 72 h	Human colon cancer cell line LoVo	Treatment with curcumin decreased p-AKT levels without affecting the total AKT	2014	[66]
	♦ 25 μΜ curcumin for 2 h prior to EGF stimulation ♦ 12.5, 25, and 50 μΜ curcumin for 6 h	HCT116 CRC cells	♦ Curcumin inhibited EGF-stimulated PI3K-Akt-mTOR signaling ♦ Treatment with curcumin reduced mTOR and p-p60S6K expression dose-dependently, whereas p-AKT levels were increased, possibly through the inhibition of PHLPP	2009	[67]
Glioblastoma	5, 30, and 50 μΜ curcumin for 6 h	U138MG, U87, and U373 GBM cells	Treatment with curcumin decreased p-AKT levels dose-dependently	2012	[68]
	♦ 20 μΜ curcumin for 24 h ♦ 10 and 20 μΜ curcumin for 24 h	U87 and U251 GBM cells	♦ Treatment with curcumin decreased the phosphorylation of AKT (p-AKT) and mTOR (p-mTOR) in U251 cells ♦ Treatment with 10 or 20 μΜ curcumin increased PTEN expression	2020	[69]
	25 μΜ curcumin for 24 h	U87MG cells	Treatment with curcumin resulted in decreased levels of PI3Kp85, pPI3Kp85, total Akt, p-Akt, mTOR, and p-mTOR	2019	[57]
	10, 15, and 20 μΜ curcumin for 48 h	SNB19 and A1207 cells	Treatment with curcumin decreased p-AKT through the targeting of NEDD4	2017	[70]
Leukemia	10, 20, and 40 μΜ curcumin for 24 h	B-Pre-ALL cells (697, REH, RS4;11, and SupB15 cells)	Treatment with curcumin dose-dependently decreased the protein expression of p-AKT, AKT, p-GSK3, GSK3, and p-FOXO1 FOXO1	2019	[61]
	20 and 40 μΜ curcumin for 24 h	T-ALL cells (CEM, HSB2, Jurkat and Molt-4)	Treatment with curcumin resulted in the dephosphorylation of AKT and, thus, inactivation of its downstream effectors, FOXO and GSK3	2006	[71]
	30 μM curcumin for 3, 6, 12, and 24 h or 10, 20, and 40 μM curcumin for 24 h	Human Ph+ B-ALL cell line SUP-B15	Treatment with curcumin dose- and time-dependently decreased the expression of p-AKT, p-mTOR, p-GSK3, and p-P70S6, but had no effect on PTEN	2015	[72]
	25 μΜ curcumin for 24 h	AML cell lines (HL-60, ML-2, MOLM-13, OCI-AML3, OCI-AML5, and U937)	Treatment with curcumin decreased the protein expression of p-AKT in OCI-AML5 and ML-2 cells	2021	[73]
Lung	40 μΜ curcumin for 48 h	NSCLC A549 cells	Treatment with curcumin resulted in dose-dependent inhibition of Akt phosphorylation and mTOR phosphorylation	2018	[56]
	20 μΜ curcumin for 24 h	NSCLC A549 cells	Treatment with curcumin decreased the levels of p-AKT and p-mTOR without affecting the total AKT and total mTOR	2020	[74]
	5, 10, and 20 μΜ curcumin for 24 h	NSCLC A549 cells	Treatment with curcumin at higher concentrations decreased the levels of PI3K and AKT	2015	[75]
	25, 50, and 100 μΜ curcumin for 48 h	NSCLC A549 cells	Treatment with curcumin at higher concentrations decreased the levels of p-AKT	2020	[76]
Prostate	5, 10, 15, and 20 μΜ curcumin for 24 h	LNCaP, DU145, and PC3 cells	Treatment of LNCaP cells with curcumin reduced the expression of PI3K subunits (p110 and p85) and p-AKT	2007	[77]
	♦ 10, 20, 30, 40, and 50 μΜ curcumin for 1 h ♦ 40 μΜ curcumin for up to 120 h	PC-3 cells	♦ Treatment with curcumin inhibited the levels of p-Akt (T308 and S473), p-FoxO1 (S256), p-GSK3β (S9), p-mTOR (S2448/2481), p-p70S6K (T389), and p-S6 (S235/236) dose-dependently ♦ Treatment with curcumin inhibited the levels of the above mentioned proteins time-dependently	2008	[78]
Thyroid	20 μΜ for 48 h	FTC133 cells	Treatment with curcumin decreased PI3K and p-Akt protein expression	2014	[79]
	12.5, 25, and 50 μΜ curcumin for 24 h	BCPAP cells	Treatment with curcumin reduced the levels of phosphorylated PDK1, AKT, p70S6, p85S6, S6, and 4E-BP1 without affecting the total expression of the corresponding proteins	2022	[80]
	12.5, 25, and 50 μΜ curcumin for 24 h	BCPAP and KTC-1 cells	Treatment with curcumin decreased the levels of p-AKT (Thr308 and Ser473), p70S6, p85S6, S6, and 4E-BP1 dose-dependently	2021	[81]
